# Expanding Diabetes Screening to Identify Undiagnosed Cases Among Emergency Department Patients

**DOI:** 10.5811/westjem.59957

**Published:** 2023-08-08

**Authors:** David C. Lee, Harita Reddy, Christian A. Koziatek, Noah Klein, Anup Chitnis, Kashif Creary, Gerard Francois, Olumide Akindutire, Robert Femia, Reed Caldwell

**Affiliations:** * New York University, NYU Grossman School of Medicine, Ronald O. Perelman Department of Emergency Medicine, New York, New York; † New York University, NYU Grossman School of Medicine, Department of Population Health, New York, New York; ‡ Bellevue Hospital Center, Department of Emergency Medicine, New York, New York

## Abstract

**Introduction:**

Diabetes screening traditionally occurs in primary care settings, but many who are at high risk face barriers to accessing care and therefore delays in diagnosis and treatment. These same high-risk patients do frequently visit emergency departments (ED) and, therefore, might benefit from screening at that time. Our objective in this study was to analyze one year of results from a multisite, ED-based diabetes screening program.

**Methods:**

We assessed the demographics of patients screened, identified differences in rates of newly diagnosed diabetes by clinical site, and the geographic distribution of high and low hemoglobin A1c (HbA1c) results.

**Results:**

We performed diabetes screening (HbA1c) among 4,211 ED patients 40–70 years old, with a body mass index ≥25, and no prior history of diabetes. Of these patients screened for diabetes, 9% had a HbA1c result consistent with undiagnosed diabetes, and nearly half of these patients had a HbA1c ≥9.0%. Rates of newly diagnosed diabetes were notably higher at EDs located in neighborhoods of lower socioeconomic status.

**Conclusion:**

Emergency department-based diabetes screening may be a practical and scalable solution to screen high-risk patients and reduce health disparities experienced in specific neighborhoods and demographic groups.

## INTRODUCTION

Among the 35 million Americans with diabetes, nearly one in four are unaware of their condition.^1^ Diabetes screening traditionally occurs in primary care settings, but many people at high risk of diabetes face substantial barriers when accessing healthcare.^2^^,^^3^ Therefore, diagnosis is delayed among those most likely to develop diabetes at an early age, and there is greater risk of diabetes-related morbidity and mortality that would otherwise have been preventable.^4^^,^^5^ Many high-risk patients, however, do have frequent interactions with the healthcare system. Patients with socioeconomic burdens and time constraints are more likely to visit emergency departments (ED) for care.^6^^–^^8^

In June 2019, we began an ED initiative to test hemoglobin A1c (HbA1c) for patients without a history of diabetes who met US Preventive Services Task Force (USPSTF) guidelines for screening. Our goal in this study was to evaluate one year of results from this screening program to assess program efficacy and the demographics of ED patients with newly diagnosed diabetes. We aimed to examine whether ED-based diabetes screening can be scalable and effective in helping address disparities in the diagnosis of new diabetes and identifying patients who need linkage to outpatient care for management.

## METHODS

### Study Design

We performed a retrospective chart review of patients screened for diabetes from February 2021–January 2022 with a HbA1c test at four EDs in New York City. The primary outcome of interest was the percentage of patients with an elevated HbA1c. We analyzed the demographic characteristics of screened patients, differences by clinical site, and the geographic distribution of results among patients screened.

### Study Population

Patients screened included anyone already receiving blood work for their clinical care who met USPSTF guidelines for diabetes screening. At the time of the study, those guidelines recommended screening for adults 40–70 years old with a body mass index (BMI) ≥25. Eligible patients were automatically identified through the electronic health record (EHR) as not having a history of diabetes (based on problem list, auto-populated via the shared health record system Care Everywhere [Care Everywhere LLC, Natick, MA] or by patient-provided past medical history at ED triage), and as not having a HbA1c test in the previous six months. The HbA1c tests were offered at no charge, and patients were given the opportunity to decline the test, although data on those declined was not tracked.

### Statistical Analysis

We performed a descriptive analysis of screened patients during the study period, for both the overall study population and by clinical site. We analyzed BMI categorically: overweight (BMI 25.0–29.9); obese (30.0–39.9); and morbidly obese (≥ 40). The HbA1c result was categorized as normal (<5.7%), prediabetes (5.7%–6.4%), diabetes (6.5%–8.9%), and poorly controlled diabetes (≥9.0%). To identify statistically significant differences in patient characteristics among hospitals, we used ANOVA for continuous variables and chi-squared tests for categorical variables.

We conducted a geospatial analysis to determine whether high and low HbA1c values were concentrated in specific geographic areas. To do this, we performed a Getis-Ord Gi* hotspot analysis on HbA1c values, a commonly used approach to identify significant spatial clustering of high and low values for a given variable.^9^^,^^10^ We used the K-nearest neighbors to model spatial proximity.

Statistical analyses were performed using Stata 16.1 (Stata Corp, College Station, TX). Geographic analysis was performed using ArcGIS Pro 2.8.3 (ESRI; Redlands, CA). This study was approved by the institutional review board at New York University School of Medicine.

## RESULTS

### Study Population

We screened 4,211 ED patients 40–70 years old, with BMI ≥25 and no prior history of diabetes (Table 1). Of the patients screened, 58% were minorities; the proportion of each race/ethnicity differed among the four sites. Demographic differences at each site generally reflected the local populations living near those EDs. Of the patients screened, 16% reported that English was not their primary language. By insurance type, 55% of patients screened had private/commercial insurance, 15% were insured by Medicare, 25% by Medicaid, and 5% were self-pay or uninsured. Thirty-four percent of patients screened at NYU Brooklyn were insured by Medicaid, much higher than the proportion at the other EDs. In terms of BMI, 43% were obese, 10% were morbidly obese, and the remainder were overweight, given the BMI screening cutoff of 25.

**Table 1.** Characteristics of emergency department patients screened for diabetes and hemoglobin A1c results.

Population characteristics	All hospitals	NYU-Tisch Hospital Manhattan	NYU Brooklyn	NYU Cobble Hill	NYU Long Island	*P*-value for differences
Patients	4,311	1,092	1,079	589	1,551	
Age (median)	54	55	53	51	55	< 0.01
Gender						
Female	2,155 (50%)	513 (47%)	496 (46%)	330 (56%)	807 (52%)	< 0.01
Male	2,156 (50%)	579 (53%)	583 (54%)	259 (44%)	744 (48%)	
Race/ethnicity						
White	1,811 (42%)	491 (45%)	345 (32%)	194 (33%)	776 (50%)	< 0.01
Black	905 (21%)	229 (21%)	129 (12%)	224 (38%)	310 (20%)	
Hispanic	1,121 (26%)	218 (20%)	464 (43%)	112 (19%)	357 (23%)	
Asian	129 (3%)	33 (3%)	32 (3%)	12 (2%)	47 (3%)	
Other	345 (8%)	120 (11%)	108 (10%)	47 (8%)	62 (4%)	
Language						
English	3,621 (84%)	1,005 (92%)	712 (66%)	565 (96%)	1,334 (86%)	< 0.01
Spanish	517 (12%)	44 (4%)	281 (26%)	18 (3%)	186 (12%)	
Other	172 (4%)	44 (4%)	86 (8%)	6 (1%)	62 (2%)	
Insurance						
Private	2,371 (55%)	612 (56%)	432 (40%)	342 (58%)	977 (63%)	< 0.01
Medicare	733 (17%)	229 (21%)	183 (17%)	88 (15%)	248 (16%)	
Medicaid	1,035 (24%)	240 (22%)	367 (34%)	141 (24%)	295 (19%)	
Self-pay	172 (4%)	22 (2%)	97 (9%)	18 (3%)	31 (2%)	
BMI						
Overweight	2,026 (47%)	557 (51%)	529 (49%)	253 (43%)	698 (45%)	< 0.01
Obese	1,854 (43%)	437 (40%)	453 (42%)	253 (43%	713 (46%)	
Morbidly obese	431 (10%)	98 (9%)	97 (9%)	82 (14%)	140 (9%)	
HbA1c result						
≥ 9.0%	172 (4%)	22 (2%)	97 (9%)	24 (4%)	31 (2%)	< 0.01
6.5% to 8.9%	216 (5%)	44 (4%)	76 (7%)	29 (5%)	62 (4%)	
5.7% to 6.4%	776 (18%)	164 (15%)	183 (17%)	88 (15%)	326 (21%)	
< 5.7%	3,147 (73%)	863 (79%)	723 (67%)	448 (76%)	1,132 (73%)	

*BMI*, body mass index; *HbA1c*, hemoglobin A1c.

### Primary Outcome

Eighteen percent of screened ED patients had a HbA1c result consistent with prediabetes, and 9% had HbA1c results consistent with undiagnosed diabetes. Notably, almost half of ED patients with a new diagnosis of diabetes had a HbA1c ≥9.0%, consistent with poorly controlled diabetes. Comparing the proportion of patients newly diagnosed with diabetes at each of the four EDs, rates were notably higher at NYU Brooklyn (16%) and NYU Cobble Hill (9%) compared to NYU-Tisch Hospital in Manhattan (6%) and NYU Long Island (6%) (*P*-value < 0.01).

### Geospatial Analysis of HbA1c Results

**Figure 1.**
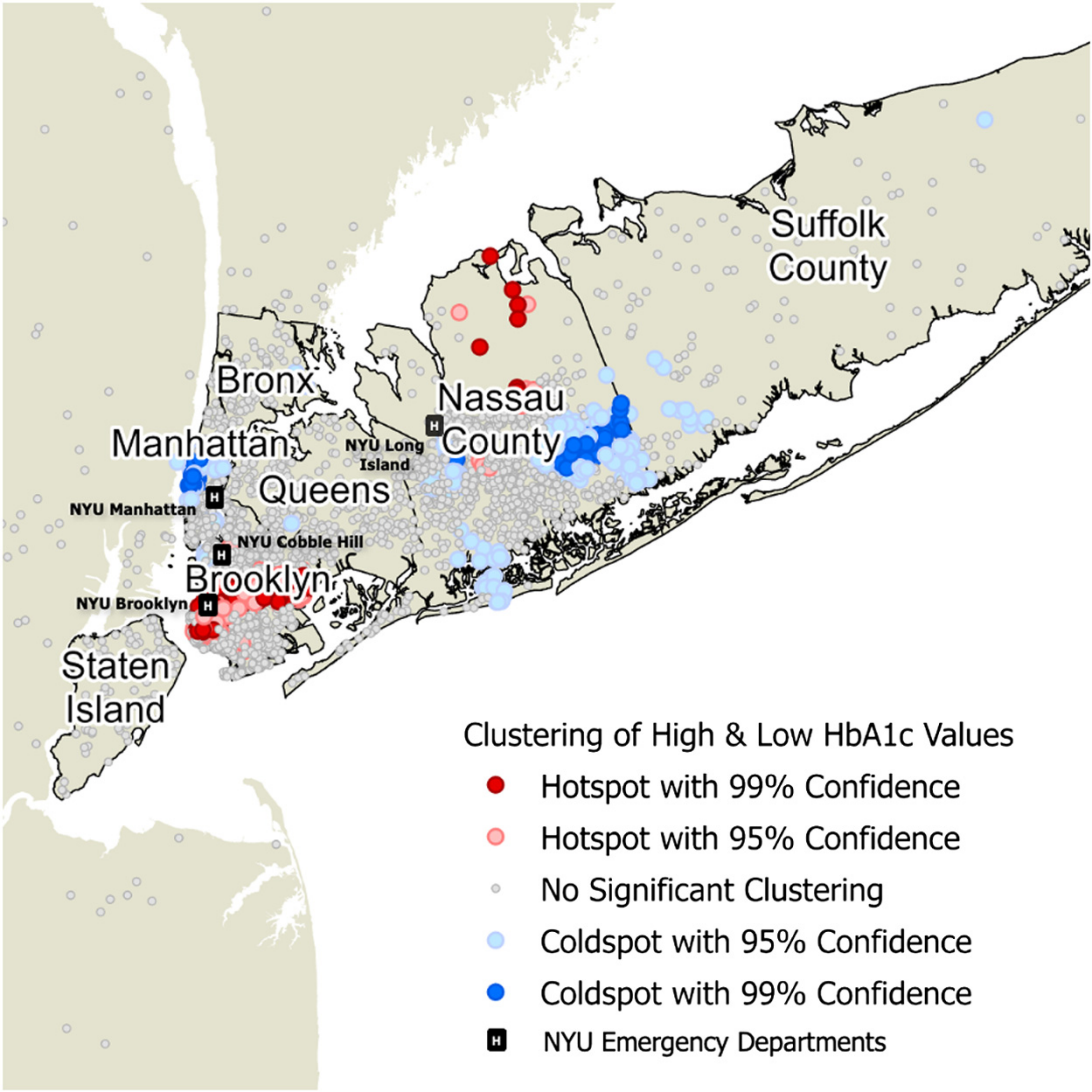
Geographic distribution of emergency department patients screened for diabetes in the New York City area and marked locations of the emergency departments.

In a geospatial analysis, we identified statistically significant clustering of high and low HbA1c values in the New York City area (Figure 1). Sensitivity analyses for the number of nearest neighbors demonstrated a generally similar geographic location of these hot and cold spots, which suggests that these findings were robust (not affected by changing the number of nearest neighbors specified).^11^

## DISCUSSION

Our study reviewed one year of data from an ED-based diabetes screening program and found a high rate of previously undiagnosed diabetes among ED patients meeting USPSTF criteria to be screened. Many of these patients had poorly controlled diabetes (HbA1c of >9.0%). While EDs are not designed to provide long-term management of chronic diseases, rates of undiagnosed diabetes are known to be two times higher among patients with poor access to primary care.^12^ Patients diagnosed with diabetes through preventive screening are diagnosed much earlier, and have lower mortality, when compared to patients diagnosed with diabetes due to clinical symptoms.^13^^,^^14^ The ED provides a setting for diabetes screening where new cases could be diagnosed that might otherwise go undetected, especially among high-risk patients with poor access to primary care. Previously published efforts to screen ED patients for diabetes have demonstrated even higher rates of undiagnosed diabetes, which may be due to the screening criteria used or the patient populations in those health systems.^15^^–^^19^

Our study also found higher rates of undiagnosed diabetes in our EDs that are located primarily in neighborhoods of lower socioeconomic status, including those with a high proportion of patients for whom English is not the primary language. Leveraging the ED to promote diabetes screening has the potential to reduce racial and ethnic disparities in diabetes burden, especially among patients who may face language barriers. Limiting preventive screening to the primary care setting risks the perpetuation of poor health outcomes for those patients who face barriers to healthcare access. If the primary concern is that these newly diagnosed ED patients may not follow up for appropriate outpatient care, then efforts should focus on how to improve care coordination from the ED to the primary care setting.

Our study demonstrates that the highest yield of this diabetes screening was in EDs seeing a high proportion of patients of lower socioeconomic status. In a multivariable analysis controlling for age, gender, race/ethnicity, language, and insurance type, we found male gender, all non-White race/ethnicities, other language, and self-pay insurance status were statistically significant predictors of previously undiagnosed diabetes. In addition, the location of hot and cold spots of HbA1c values correlated well with prior studies of the geographic distribution of poor glycemic control among diabetes patients in New York City.^20^ Promoting diabetes screening in EDs that serve high-risk patient populations can help identify undiagnosed cases of diabetes in these neighborhoods, which may be a critical step in addressing poor health outcomes experienced in these geographic areas.

## LIMITATIONS

Our study has several limitations. First, ED patients receiving bloodwork are likely to have some differences from patients not receiving bloodwork; these findings may not be generalizable to every ED patient. Additionally, our study was in one metro area, and while our findings do fit well with existing diabetes literature for the New York City area, it is possible that other geographic areas will have different patient types visiting the ED and, therefore, may have different amounts of undiagnosed diabetes than found in our study. It is also possible that patients identified as newly diagnosed in our study did have a previous diagnosis elsewhere, which they did not disclose during their ED visit and was not available in our EHR data. Finally, there may have been other confounders (eg, dietary behaviors) that are highly correlated with the factors analyzed in our study, which may be an unmeasured predictor of undiagnosed diabetes and may be a limitation of relying only on EHR data.

## CONCLUSION

We found a high percentage of screened patients in the ED with undiagnosed diabetes. We further found that the burden of undiagnosed diabetes in the ED is concentrated in certain geographic areas, corroborating known socioeconomic elements to undiagnosed diabetes. This suggests that ED-based diabetes screening can be a scalable solution for addressing disparities in the burden of diabetes and identifying patients who need linkage to outpatient care. Further work can focus on improved screening criteria specific to the ED population to improve yield and follow-on systems development for further care of newly diagnosed patients.
